# Genome Sequence of *Lactobacillus fermentum* Strain NCC2970 (CNCM I-5068)

**DOI:** 10.1128/genomeA.01254-16

**Published:** 2016-11-17

**Authors:** Caroline Barretto, Catherine Ngom-Bru, Alienor Genevaz, Coralie Fournier, Deborah Moine, Mohamed Kassam, Agnes Iltis, Pierre Sagory-Zalkind, Pierre-Emmanuel Ciron, Gilles Faucherand, Patrick Descombes, Stephane Duboux

**Affiliations:** a Nestec Ltd., Nestlé Research Center, Lausanne, Switzerland; b Nestlé Institute of Health Sciences, Lausanne, Switzerland; c Genostar, Montbonnot-Saint-Martin, France

## Abstract

*Lactobacillus fermentum* NCC2970 (CNCM I-5068) is a lactic acid bacterium originating from the Nestle Culture Collection. Here, we disclose its full 1.9-Gb genome sequence comprising one chromosome with no plasmid.

## GENOME ANNOUNCEMENT

*Lactobacillus fermentum* is a heterofermentative lactic acid bacterium (LAB) belonging to the *Lactobacillaceae* family within the *Bacilli* class of the *Firmicutes* phylum. The species is listed in the qualified presumption of safety (QPS) published by the European Food Safety Authority (EFSA) (1), and a previously described strain belonging to this species has been generally recognized as safe by the U.S Food and Drug Administration (GRAS notice no. 531). *L. fermentum* strains have been previously reported for their different properties, including technological (2) or probiotic functionalities (3–6). *Lactobacillus fermentum* NCC2970 originates from the Nestlé Culture Collection and has also been deposited at the National Collection of Microorganisms Cultures (CNCM) under CNCM I-5068.

Genomic DNA was extracted from mid-exponential cultures using a Gentra DNA Purgene kit (Qiagen), and 20-kb libraries were prepared following the Pacific Biosciences (PacBio) protocol and BluePippin size selection (Sage Science). Sequencing was performed on the PacBio RSII platform using P6/C4 chemistry on single-molecule real-time (SMRT) cells with a 240-min collection protocol. The subreads were *de-novo* assembled using the PacBio Hierarchical Genome Assembly Process (HGAP)/Quiver software package (7) followed by Circlator for genome circularization (8, 9) and a final polishing step with Quiver. The strain was assembled into a single contig corresponding to the chromosome. No plasmid could be detected.

During sequencing, epigenetic modifications of each nucleotide position were measured as kinetic variations (KVs) in nucleotide incorporation rates. Motifs were deduced from the KV data (10). Analysis was performed using the SMRT portal RS_Modification_and_Motif_Analysis protocol (PacBio).

The length of the chromosome of *L. fermentum* NCC2970 (CNCM I-5068) is 1,949,874 bp, with a GC content of 52.21%. Automated expert annotation carried out by Genostar using a proprietary pipeline (2) revealed 5 rRNA operons and 56 tRNA genes. It indicated 1,927 protein-coding sequences on the chromosome, of which 1,650 (86%) were annotated with known biological functions and 277 (14%) encode hypothetical proteins or uncharacterized proteins.

The *L. fermentum* NCC2970 (CNCM I-5068) genome has a similar size and structure than other publically available *L. fermentum* genomes (F-6 [5], CECT 5716 [6]). It contains, however, some specific features, especially with regard to its substrate usage capacity. Compared to other *L. fermentum* genomes, it harbors a more complete shikimate pathway with a 3-dehydroquinate dehydratase (EC 4.2.1.10; CDS0787) and a shikimate dehydrogenase (EC 1.1.1.25; CDS0788), which are, respectively, the third and the fourth enzymes of this pathway, enabling the conversion of quinic acid to shikimic acid. It harbors also a 5′-nucleotidase (EC 3.1.3.5; CDS0645), which in *C. glutamicum* is required for growth when nucleotides are provided as the sole source of phosphate (11), as well as a phosphopentomutase (EC 5.4.2.7; CDS0031), which has been shown in *Bacillus cereus* to be necessary to isomerize ribose-1P in ribose-5P, thus enabling it to enter the pentose phosphate pathway (12).

### Accession number(s).

This whole-genome project has been deposited at DDBJ/EMBL/GenBank under the accession number CP017151. The version described in this paper is the first version.
